# Efficacy and Safety of Immunosuppressive Monotherapy Agents for IgA Nephropathy: A Network Meta-Analysis

**DOI:** 10.3389/fphar.2020.539545

**Published:** 2021-01-22

**Authors:** Shisheng Han, Tianwen Yao, Yan Lu, Min Chen, Yanqiu Xu, Yi Wang

**Affiliations:** Department of Nephrology, Yueyang Hospital of Integrated Traditional Chinese and Western Medicine, Shanghai University of Traditional Chinese Medicine, Shanghai, China

**Keywords:** immunosuppressive therapy, IgA nephropathy, network meta-analysis, systematic review, renoprotective

## Abstract

**Background:** The efficacy and safety of immunosuppressive monotherapy agents were evaluated for immunoglobulin A nephropathy (IgAN) using a network meta-analysis approach.

**Methods:** Randomized controlled trials (RCTs) published prior to October 1, 2019, using immunosuppressive agents for treating IgAN, were systematically searched in PubMed, Embase, Cochrane Library, and Web of Science databases. Relative risks (RRs) or standard mean differences with 95% confidence intervals (CIs) were estimated using the random-effects model. The primary outcomes were clinical remission, end-stage renal disease (ESRD), and serious adverse events (SAEs). The secondary outcomes were urinary protein excretion and serum creatinine.

**Results:** Twenty-five RCTs with 2,005 participants were deemed eligible. Six medications were evaluated: corticosteroids, mycophenolate mofetil (MMF), tacrolimus (TAC), cyclosporine, leflunomide, and hydroxychloroquine (HCQ). Steroids (RR 1.50, 95% CI 1.17–1.93), MMF (RR 2.05, 95% CI 1.15–3.65), TAC (RR 3.67, 95% CI 1.06–12.63), and HCQ (RR 3.25, 95% CI 1.05–10.09) significantly improved clinical remission rates compared to supportive care alone. Only steroids reduced the risk of ESRD (RR 0.35, 95% CI 0.12–0.98); however, there were significantly more SAEs than in the control group (RR 2.90, 95% CI 1.37–6.13). No significantly different effects in serum creatinine levels were found among the therapies. MMF showed no significant improvement in remission when excluding studies with a follow-up of fewer than 2 years in the sensitivity analysis (RR 1.41, 95% CI 0.40–4.92). The effect of TAC in the decrease of proteinuria was reversed after discontinuing medication for 3 months; the long-term effects of HCQ could not be evaluated due to the short follow-up duration.

**Conclusion:** Corticosteroids might induce remission and increase renal survival in IgAN; however, adverse reactions should be taken into consideration. MMF, TAC, and HCQ might improve the remission of proteinuria when treating IgAN, but showed no superiority compared to steroids, and the long-term effects require further study.

## Introduction

Immunoglobulin A nephropathy (IgAN) is one of the most common glomerular diseases and a leading cause of end-stage renal disease (ESRD) worldwide (Rodrigues et al., 2017). Approximately 20–40% of patients progress to ESRD within 10–20 years after diagnosis (Magistroni et al., 2015). Currently, the inhibition of the renin-angiotensin system (RAS) for supportive care is the preferred treatment for IgAN and corticosteroids are recommended for patients with proteinuria greater than 1.0 g/24 h and an estimated glomerular filtration rate (eGFR) greater than 50 ml min^−1^·1.73 m^2^, despite the fact that standard therapies are prescribed according to the Kidney Disease Improving Global Outcomes (KDIGO) guidelines (Radhakrishnan and Cattran, 2012). However, in light of the KDIGO Controversies Conference in 2017, this recommendation may need to be revisited (Floege et al., 2019). More recently, steroids were shown to be potentially beneficial for clinical remission of IgAN, despite being associated with a significant increase in serious adverse events (SAEs) (Lv et al., 2017). Corticosteroid-based immunosuppressive treatments have also been used as a potential treatment for remission in IgAN; however, they are also limited by their high risks of adverse events (Rauen et al., 2015). Immunosuppressive monotherapy is an optional treatment for IgAN and includes calcineurin inhibitors and mycophenolate mofetil (MMF) (Zhang and Zhang, 2018). Previous pairwise meta-analyses indicated that calcineurin inhibitors and MMF could be effective in the treatment of IgAN (Peng et al., 2016; Du et al., 2017), although their independent effects are controversial and their relative effects among different immunosuppressive agents are unknown. As network meta-analysis (NMA) can compare the effects of all these drugs under a coherent framework, in addition to the probability of optimal treatment, we conducted an NMA to determine the effect of monotherapy for IgAN using different immunosuppressive agents and, if possible, predicted the best candidate for treatment.

## Methods

### Design and Registration

This work was performed according to the Preferred Reporting Items for Systematic Reviews and Meta-Analyses (PRISMA) extension statement for NMA (Hutton et al., 2015) (see Supplementary Table S1). The NMA protocol was registered in PROSPERO: CRD42019147935.

### Search Strategy

Eligible studies published prior to October 1, 2019, were searched through PubMed, Embase, the Cochrane Central Register of Controlled Trials (CENTRAL), and the Web of Science. Medical subject headings and all-fields searches consisted of three parts without language restrictions: immunosuppressive agent (cyclophosphamide [CYC], azathioprine [AZA], cyclosporine [CsA], tacrolimus [TAC], leflunomide (LEF), hydroxychloroquine [HCQ], MMF, and steroid), IgAN, and randomized controlled trial (RCT). The search strategies are shown in Supplementary Text 1. The related systematic reviews and meta-analyses on immunosuppressive agents for IgAN were also checked (Peng et al., 2016; Du et al., 2017; Qian et al., 2019; Zhang et al., 2019).

### Eligibility Criteria

The inclusion criteria were as follows: 1) participants: patients with biopsy-proven IgAN; 2) different immunosuppressive monotherapy agents: CYC, AZA, MMF, CsA, TAC, LEF, HCQ, or steroids that were compared with each other or with non-immunosuppressive treatments. In addition, supportive therapies that were administered to both groups; 3) outcomes: the primary outcomes were clinical remission, including complete remission (CR), or partial remission (PR), which were provided in each original study, and the endpoint of the ESRD and SAEs, including death, serious infection, new diabetes mellitus, and other SAEs defined by the original studies, and the secondary outcomes included urinary protein excretion and serum creatinine; and 4) study design: RCTs.

The exclusion criteria included: 1) secondary IgAN; or 2) no data available for analysis.

### Study Selection and Data Extraction

**FIGURE 1:**
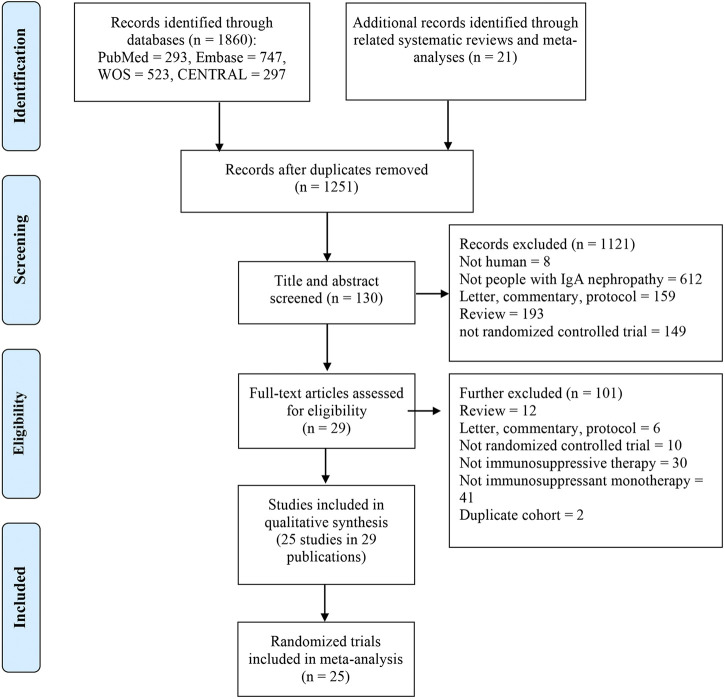
Flow chart of the study selection.

Two investigators (S.-S. Han and T.-W. Yao) performed the study selection process and extracted the data independently and any disagreement was solved via discussion with a third reviewer (Y. Wang). The process of study selection is summarized in the PRISMA flowchart (Figure 1). The following data were extracted: first author, year of publication, location, baseline information for all groups (sample size, age, and sex), intervention details, duration of follow-up, loss to follow-up, and outcomes.

### Risk of Bias Assessment

The risk of bias was assessed by the Cochrane Handbook (version 5.1.0) for each trial by two investigators independently (Y. Lu and M. Chen). This tool consists of seven items—the assessments of selection bias, performance bias, detection bias, attrition bias, reporting bias, and other bias, with each domain at three levels—low risk, high risk, and unclear risk (Higgins et al., 2011).

### Statistical Analysis

All statistical analyses were performed using the Stata software (version 14.0) and the network command (White, 2015) utilizing previously reported routines (Chaimani et al., 2013). Continuous data were compared using standardized mean differences (SMDs) and corresponding 95% confidence intervals (CIs) because they could be detected at different follow-up times. Relative risks (RRs) with 95% CIs were calculated for discrete data. Random-effects models of pairwise meta-analyses were performed for each outcome of direct contrast. The heterogeneity was estimated by a common heterogeneity variance (tau). Values of 0.1–0.5, 0.5–1.0, and >1.0 represented low, high, and extreme heterogeneity, respectively (Turner et al., 2012). An inconsistency test (based on the design-by-treatment interaction approach) was applied to evaluate the inconsistency of the entire network (Higgins et al., 2012). In the case in which there was no inconsistency, a random-effects NMA was performed to compare all the interventions for each predefined outcome in the frequentist framework. The surface under the cumulative ranking curve (SUCRA) value was used to rank the medications. A sensitivity analysis was conducted by excluding studies with follow-ups of fewer than 2 years. A subgroup analysis was performed by stratifying the groups according to the eGFR and histological lesions to clarify potential variables related to immunosuppressive therapy responsiveness and renal outcomes. The small-study effect was assessed by a comparison-adjusted funnel plot.

## Results

### Characteristics of the Included Studies

**FIGURE 2:**
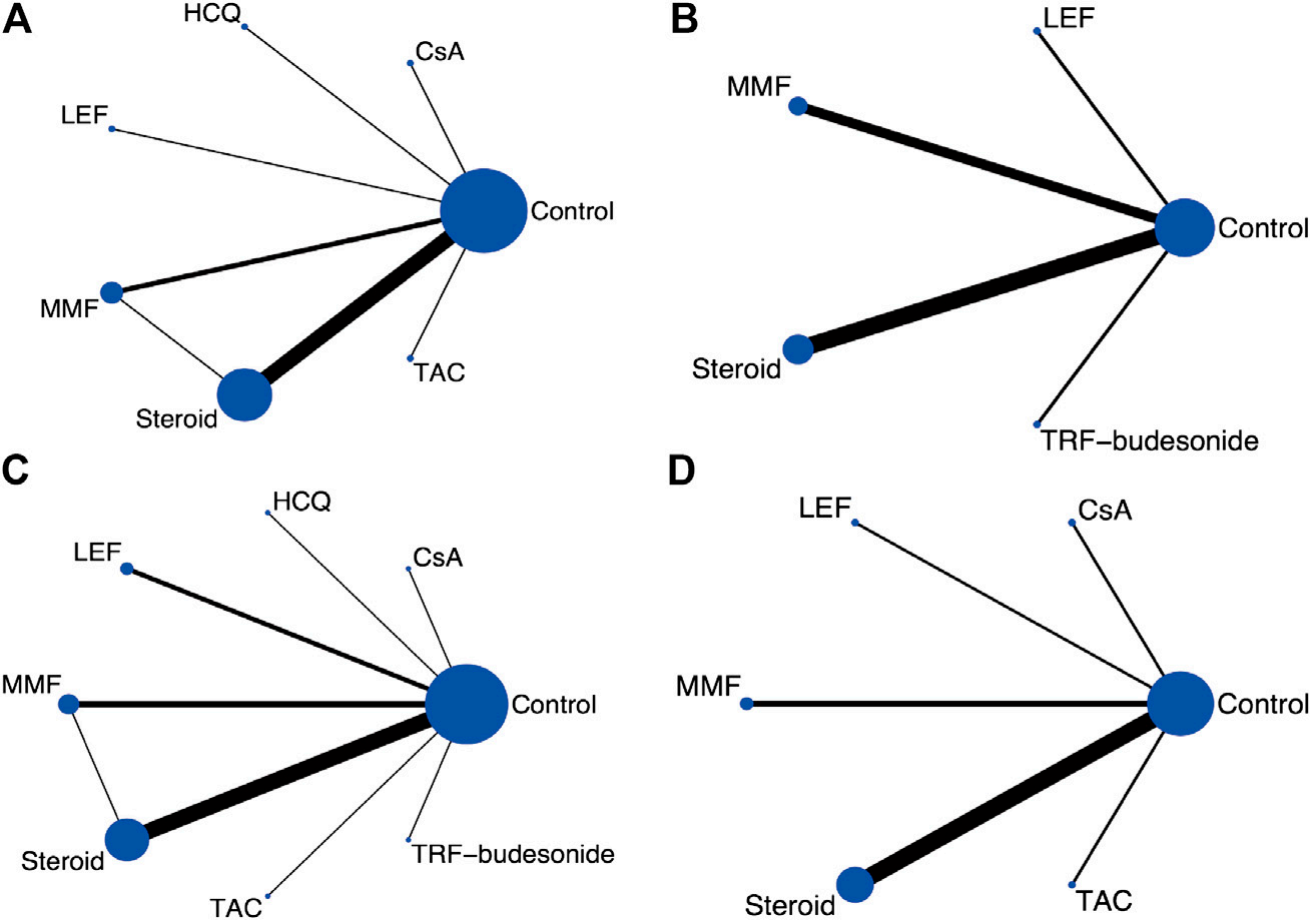
Network graphs for the predefined outcome **(A)** Clinical remission **(B)** ESRD **(C)** Serious adverse events **(D)** Serum creatinine. (CsA: Cyclosporine; ESRD: End-stage renal disease; HCQ: Hydroxychloroquine; LEF: Leflunomide; MMF: Mycophenolate mofetil; TRF: Targeted-release formulation; TAC: Tacrolimus).

One thousand two hundred and fifty-one publications were retrieved after removing duplications. From a total of 29 publications, 25 RCTs were identified for meta-analyses (Lai et al., 1986; Lai et al., 1987; Julian and Barker, 1993; Pozzi et al., 1999; Shoji et al., 2000; Locatelli et al., 2001; Chen et al., 2002; Katafuchi et al., 2003; Lee et al., 2003; Maes et al., 2004; Pozzi et al., 2004; Frisch et al., 2005; Kim et al., 2005; Tang et al., 2005; Hogg et al., 2006; Lou et al., 2006; Koike et al., 2008; Lv et al., 2009; Manno et al., 2009; Tang et al., 2010; Kim et al., 2013; Cheng et al., 2015; Hogg et al., 2015; Wu et al., 2016; Fellstrom et al., 2017; Lv et al., 2017; Yu et al., 2017; Tang et al., 2018; Liu et al., 2019) (Figure 1). There were 2,005 participants (1,195 males and 810 females) enrolled in the 25 RCTs. In these studies, six immunosuppressive agents, including corticosteroids, MMF, TAC, CsA, LEF, and HCQ, were reported. A novel targeted-release formulation of the steroid (targeted-release formulation (TRF)-budesonide) was included; therefore, it was presented separately to differentiate it from conventional formulations of steroids. Supportive therapies were administrated to both groups and comparisons were performed between immunosuppressive drugs and controls (placebo, 11; supportive care, 12; dipyridamole, 1), except one that compared MMF with prednisone (Chen et al., 2002). The network graphs are shown in Figure 2. The median duration of each drug was as follows: steroids, 8.5 (minimum 4, maximum 24) months; MMF, 12 (minimum 6, maximum 36) months; TAC, 16 weeks; CsA, 12 weeks; LEF, 6 (minimum 6, maximum 24) months; and HCQ, six months. The median follow-up was as follows: steroids, 35 (minimum 12, maximum 120) months; MMF, 24 (minimum 18, maximum 72) months; TAC, five years; CsA, 24 weeks; LEF, 7 (minimum 6, maximum 24) months; and HCQ, six months. Characteristics of the selected RCTs are presented in Supplementary Table S2.

### Risk of Bias Evaluation

**FIGURE 3:**
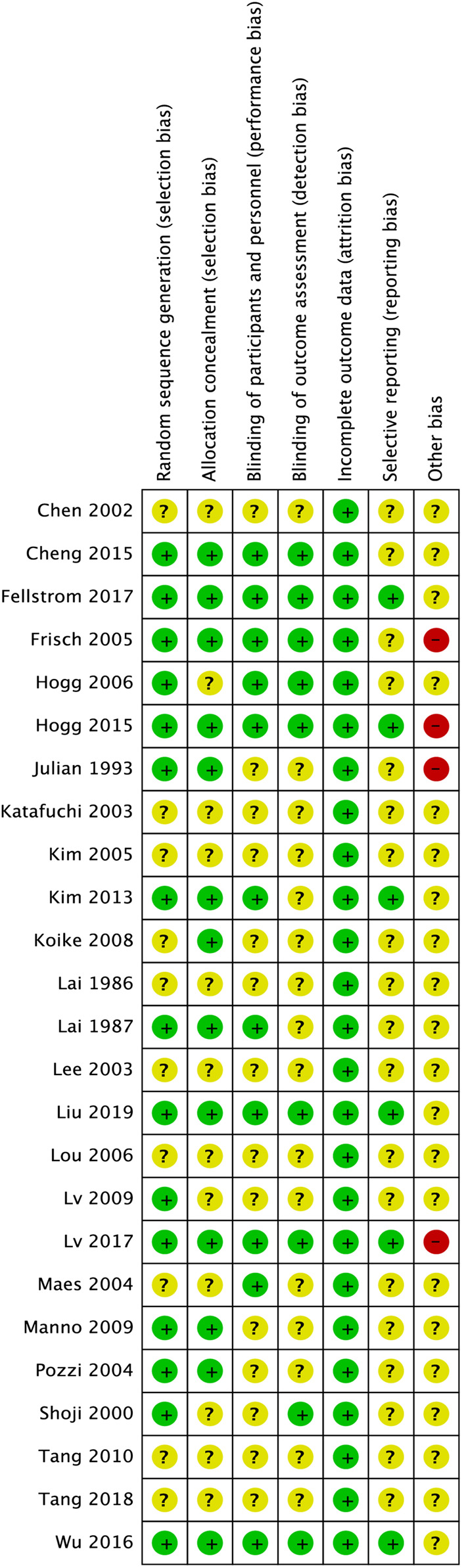
Risk of bias assessment for included studies. The green circles with “+” indicate a low risk of bias, yellow circles with “?” indicate an unclear risk of bias, and red circles with “-” indicate a high risk of bias.

Fifteen of the 25 (60%) trials described their procedures of random sequences generation appropriately and were considered to have low risk selection bias (Lai et al., 1987; Julian and Barker, 1993; Pozzi et al., 1999; Shoji et al., 2000; Frisch et al., 2005; Hogg et al., 2006; Lv et al., 2009; Manno et al., 2009; Kim et al., 2013; Cheng et al., 2015; Hogg et al., 2015; Wu et al., 2016; Fellstrom et al., 2017; Lv et al., 2017; Liu et al., 2019). Eleven (44%) trials used placebo-controlled blinding; thus, their performance bias risk was considered low (Lai et al., 1987; Maes et al., 2004; Frisch et al., 2005; Hogg et al., 2006; Kim et al., 2013; Cheng et al., 2015; Hogg et al., 2015; Wu et al., 2016; Fellstrom et al., 2017; Lv et al., 2017; Liu et al., 2019). Thirteen (52%) trials reported the processes for allocation of concealment (Lai et al., 1987; Julian and Barker, 1993; Pozzi et al., 1999; Frisch et al., 2005; Koike et al., 2008; Manno et al., 2009; Kim et al., 2013; Cheng et al., 2015; Hogg et al., 2015; Wu et al., 2016; Fellstrom et al., 2017; Lv et al., 2017; Liu et al., 2019). Nine (36%) studies reported the assessment of a blinded outcome, and their detection bias was classified as low risk (Shoji et al., 2000; Frisch et al., 2005; Hogg et al., 2006; Cheng et al., 2015; Hogg et al., 2015; Wu et al., 2016; Fellstrom et al., 2017; Lv et al., 2017; Liu et al., 2019). All (100%) studies possessed complete data. Six (24%) studies had a low reporting bias because of early registration (Kim et al., 2013; Hogg et al., 2015; Wu et al., 2016; Fellstrom et al., 2017; Lv et al., 2017; Liu et al., 2019). Three (12%) trials were terminated early (Frisch et al., 2005; Hogg et al., 2015; Lv et al., 2017), and one (4%) was a preliminary analysis (Julian and Barker, 1993); thus, other biases were classified as high risk (Figure 3).

### Primary Outcomes

Seventeen studies reported CR or PR. The NMA results indicated that steroids (RR 1.50, 95% CI 1.17–1.93), MMF (RR 2.05, 95% CI 1.15–3.65), TAC (RR 3.67, 95% CI 1.06–12.63), and HCQ (RR 3.25, 95% CI 1.05–10.09) significantly improved the clinical remission rate in patients with IgAN compared to the control group, as did the results of the pairwise meta-analyses. However, no significant differences were found in the clinical remission rates among these immunosuppressive agents for patients with IgAN (Table 1).

**TABLE 1:** Results of the meta-analysis for clinical remission.

Hydroxychloroquine						3.25 (1.19, 8.83), N = 1
3.32 (0.89, 12.37)	Leflunomide					0.98 (0.65, 1.46), N = 1
0.42 (0.02, 9.37)	0.13 (0.01, 2.46)	Cyclosporine				7.70 (0.45, 131.36), N = 1
0.89 (0.17, 4.74)	0.27 (0.07, 1.09)	2.10 (0.09, 48.52)	Tacrolimus			**3.67 (1.20, 11.19), N = 1**
1.59 (0.44, 5.67)	0.48 (0.20, 1.16)	3.76 (0.20, 71.46)	1.79 (0.46, 7.02)	Mycophenolate mofetil	1.25 (0.57, 2.74), N = 1	**2.25 (1.23, 4.13), N = 3, *p* = 0.65**
2.16 (0.68, 6.90)	0.65 (0.32, 1.33)	5.13 (0.28, 92.89)	2.44 (0.69, 8.62)	1.36 (0.75, 2.48)	Steroids	**1.52 (1.16, 1.98), N = 9, *p* < 0.01**
**3.25 (1.05, 10.09)**	0.98 (0.50, 1.90)	7.70 (0.43, 138.03)	**3.67 (1.06, 12.63)**	**2.05 (1.15, 3.65**)	**1.50 (1.17, 1.93)**	Control

The results of the network meta-analysis (bottom left) and pairwise meta-analysis (upper right) for clinical remission. Data are shown as relative risk (95% confidence interval [CI]). The risk estimate is for the column-defining treatment compared to the row-defining treatment. Statistical significance is defined as 95% CIs that do not overlap one (bold text). N = number of studies; P = p-value for heterogeneity of pairwise meta-analysis. The inconsistency and heterogeneity in the network analysis were low (p = 0.83, tau = 0.28).

Ten studies, involving LEF, MMF, steroids, and TRF-budesonide, reported the hard endpoint of ESRD. No significant differences in the incidences of ESRD were determined among these groups, except the conventional steroids group (RR 0.35, 95% CI 0.12–0.98), which showed better renal survival than the control group (Table 2).

**TABLE 2:** Results of the meta-analysis for end-stage renal disease.

TRF-budesonide				NS
5.61 (0.03, 1061.66)	Leflunomide			0.09 (0.01, 1.62), N = 1
0.59 (0.01, 45.96)	0.11 (0.00, 3.36)	Mycophenolate mofetil		0.96 (0.16, 5.72), N = 3, *p* = 0.05
1.47 (0.02, 106.16)	0.26 (0.01, 7.61)	2.50 (0.46, 13.67)	Steroids	**0.37 (0.18, 0.79), N = 5, *p* = 0.51**
0.51 (0.01, 32.36)	0.09 (0.00, 2.24)	0.86 (0.23, 3.25)	**0.35 (0.12, 0.98)**	Control

The results of network meta-analysis (bottom left) and pairwise meta-analysis (upper right) for end-stage renal disease. Data are shown as relative risk (95% confidence interval [CI]). Statistical significance is shown in bold text. N = number of studies; P = p-value for heterogeneity of pairwise meta-analysis. NS: the event in both groups were zero.

SAEs were reported in 22 studies for all included medications for IgAN. The results showed that patients with IgAN receiving steroids had higher risks of SAEs than the control group (RR 2.90, 95% CI 1.37–6.13) and similar results were observed in the pairwise meta-analysis (RR 4.27, 95% CI 1.79–10.18). All the remaining immunosuppressive agents studied showed no significant difference when compared to the controls or each other (Table 3).

**TABLE 3:** Results of the meta-analysis for serious adverse events.

TRF-budesonide							1.33 (0.37, 4.81), N = 1
1.33 (0.02, 80.00)	Hydroxychloroquine						NS
0.96 (0.20, 4.68)	0.72 (0.01, 39.14)	Leflunomide					1.42 (0.54, 3.74), N = 3, *p* = 0.68
1.21 (0.02, 68.51)	0.91 (0.00, 212.53)	1.27 (0.02, 65.07)	Cyclosporine				NS
0.44 (0.01, 13.25)	0.33 (0.00, 49.45)	0.46 (0.02, 12.35)	0.37 (0.00, 51.81)	Tacrolimus			3.00 (0.13, 69.51), N = 1
1.62 (0.22, 11.81)	1.22 (0.02, 78.95)	1.70 (0.29, 10.08)	1.34 (0.02, 81.90)	3.65 (0.11, 119.54)	Mycophenolate mofetil	NS	0.54 (0.07, 4.18), N = 4, *p* = 0.44
0.46 (0.10, 2.03)	0.35 (0.01, 18.10)	0.48 (0.14, 1.60)	0.38 (0.01, 18.72)	1.04 (0.04, 26.20)	0.28 (0.06, 1.46)	Steroids	**4.27 (1.79, 10.18), N = 10, *p* = 0.87**
1.33 (0.37, 4.81)	1.00 (0.02, 48.82)	1.39 (0.55, 3.56)	1.10 (0.02, 50.43)	3.00 (0.13, 69.52)	0.82 (0.18, 3.75)	**2.90 (1.37, 6.13)**	Control

The results of network meta-analysis (bottom left) and pairwise meta-analysis (upper right) for serious adverse events. Data are shown as relative risk (95% confidence interval [CI]). Statistical significance is shown in bold text. N = number of studies; P = p-value for heterogeneity of pairwise meta-analysis. The inconsistency and heterogeneity in the network analysis were low (p = 0.48, tau = 0.01). NS: the event in both groups were zero.

### Secondary Outcomes

Nineteen RCTs reported urinary protein excretion. The network indicated low heterogeneity (tau = 0.43) but significant inconsistency (*p* = 0.04); therefore, the results were analyzed only using a pairwise meta-analysis. The patients with IgAN receiving steroids (SMD: −0.69, 95% CI: −0.98–-0.41), LEF (SMD: −0.58, 95% CI: −0.89–-0.27), and HCQ (SMD: −1.09, 95% CI: −1.64–-0.55) had significantly lower levels of urinary protein excretion than the control group. Patients in the MMF groups showed lower urinary protein excretion than those in the steroid treatment groups (SMD: −0.77, 95% CI: −1.28–0.25) (see Supplementary Table S3). Eleven RCTs, including five immunosuppressive agents (LEF, CsA, TAC, MMF, and steroids), reported serum creatinine levels. The serum creatinine levels among the groups showed no significant differences, either in network or pairwise meta-analyses (see Supplementary Table S4).

### Sensitivity Analysis and SUCRA

As follow-up time might influence the main outcomes, we performed a sensitivity analysis to exclude studies with less than 2-years follow-ups. The sensitivity analysis for determining clinical remission was conducted among MMF, steroids, and the control group. Steroids improved clinical remission compared to non-immunosuppressive therapy (RR 1.47, 95% CI 1.10–1.96); however, MMF showed no significant improvement on remission in the sensitivity analysis compared to controls (RR 1.41, 95% CI 0.40–4.92). The results of the ESRD were stable in this sensitivity analysis among LEF, MMF, steroids, and the control group (see Supplementary Table S5).

**FIGURE 4:**
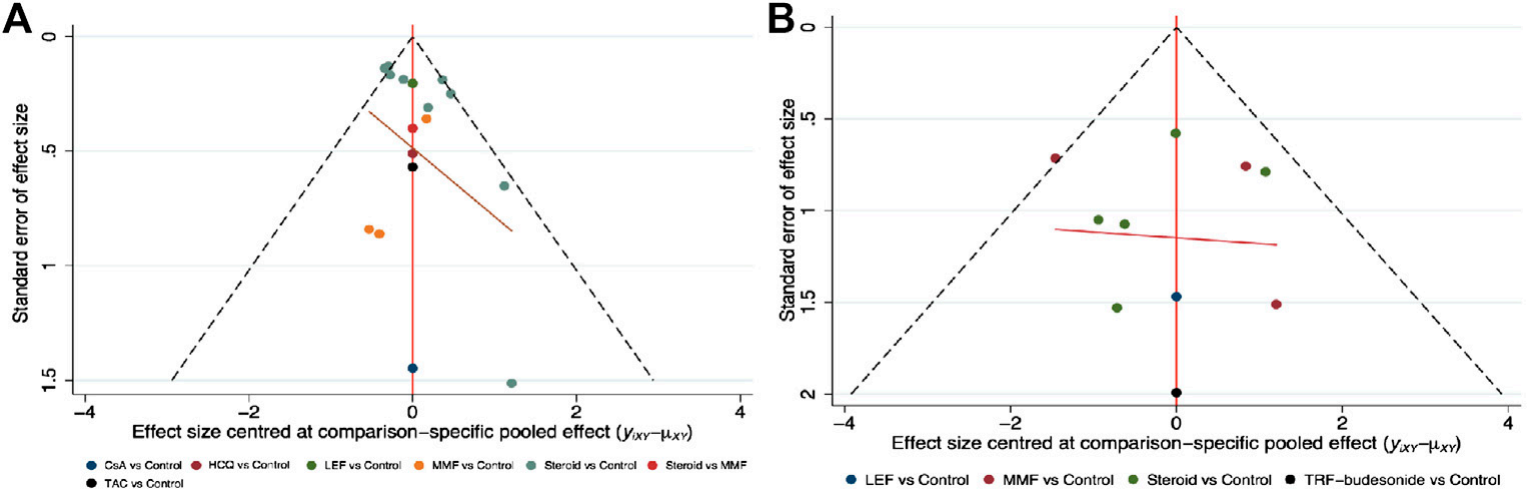
Comparison-adjusted funnel plots for clinical remission and end-stage renal disease **(A)** Clinical remission **(B)** ESRD. (CsA: Cyclosporine; ESRD: End-stage renal disease; HCQ: Hydroxychloroquine; LEF: Leflunomide; MMF: Mycophenolate mofetil; TRF: Targeted-release formulation; TAC: Tacrolimus).

Small study effects might exist according to the comparison-adjusted funnel plot (Figure 4); therefore, we next performed a sensitivity analysis to compare the main outcomes after omitting studies with less than 100 participants and the same results were documented (see Supplementary Table S6).

Considering that baseline eGFR and histological lesions might be associated with the response to immunosuppressive therapy and clinical outcomes (Nagaraju et al., 2017; Trimarchi et al., 2017), we attempted to explore the correlations by stratifying according to eGFR ≥50 ml min^−1^·1.73 m^2^ or <50 ml min^−1^·1.73 m^2^ and the Oxford classification (Trimarchi et al., 2017). Patients with eGFR <50 ml min^−1^·1.73 m^2^ showed a significantly higher risk of eGFR loss, ESRD, or death owing to renal failure than those with eGFR ≥50 ml min^−1^·1.73 m^2^ in the steroids arm (7/52 vs. 1/83, RR = 11.17, 95% CI 1.41–88.23, *p* = 0.02). Although all the included studies reported homogeneity in histological baseline, only a few studies focused on the correlations between histopathology and outcomes/therapy responsiveness. Only one among the four trials using the Oxford MEST/-C scores [MEST-C, mesangial hypercellularity (score <0.5 M0, >0.5 M1), endocapillary hypercellularity (absent E0, present E1), segmental glomerular sclerosis (absent S0, present S1), tubular atrophy/interstitial fibrosis (≤25% T0, 26–50% T1, >50% T2), and cellular/fibrocellular crescents (absent C0, ≥1 glomeruli, C1, >25% glomeruli C2)] reported the association between histological lesions and outcomes. In the steroid group, patients with E1 showed better composite outcome defined as 40% eGFR decrease, ESRD, or death than those with E0, but the differences were not significant (1/43 vs. 7/93, RR 0.31, 95% CI 0.04–2.43, *p* = 0.26). Furthermore, there were no differences between the steroid and placebo arms in the E1 or E0 scores (Lv et al., 2017). Pozzi et al. reported that the S, T, or C score had no effect on eGFR loss (Pozzi et al., 1999). However, Katafuchi et al. reported that higher E score in the steroid group was associated poor CR, and there were no differences in the M, S, T, and C scores the groups (Katafuchi et al., 2003). It should be noted that the criterion in the above two studies was not the Oxford classification.

The ranking of treatments among MMF, steroids, and controls indicated that MMF might be the best treatment to induce remission (SUCRA 91.7%), followed by steroids and controls (SUCRA 57.9 and 0.4%, respectively). Steroids were ranked as the best intervention to prevent ESRD (SUCRA 91.4%) but the worst treatment when SAEs were considered (SUCRA 3.7%, MMF 76.2%) (Table 4).

**TABLE 4:** SUCRA between steroids, mycophenolate mofetil, and control.

	Clinical remission	ESRD	Adverse events
Steroids	57.9 (15.8%)	91.4 (84.5%)	3.7 (0.1%)
Mycophenolate mofetil	91.7 (84.2%)	36.5 (14.7%)	76.2 (59.5%)
Control	0.4 (0.0%)	22.0 (0.8%)	70.1 (40.4%)

Data are shown as percentage surface under the cumulative ranking curve (possibility of optimal treatment); SUCRA = surface under the cumulative ranking curve.

## Discussion

IgAN is a common glomerulonephritis worldwide and the cause of ESRD (Moriyama, 2019). Several risk factors have correlations with the renal outcome of IgAN, of which, proteinuria has been reported as the most valuable marker for the prognosis of the treatment response, as well as deteriorated renal function (Barbour and Reich, 2018). Steroids and immunosuppressive regimens are potential optimal therapies to reduce proteinuria and improve renal survival; however, adverse events, especially serious infections, are significant (Barratt and Tang, 2018). Another choice for IgAN might be the use of immunosuppressive monotherapy agents; nevertheless, the efficacy and adverse reactions have not been determined.

This NMA was an effort to compare the direct and indirect effects of single therapy with different immunosuppressive agents, besides corticosteroids, with and without supportive care, in treating patients with IgAN. The study included 25 RCTs with 2,005 subjects, involving corticosteroids, MMF, TAC, CsA, LEF, and HCQ. The results indicated that steroids, MMF, TAC, and HCQ might improve clinical remission rates for IgAN; however, the beneficial effect of MMF for remission was not significant in studies with follow-up timepoints of more than 2 years, suggesting that the long-term efficacy of MMF for IgAN might be poor. Although TAC showed a beneficial effect in inducing remission, the 5-years follow-up of the same participants presented negative results (Yu et al., 2017). The follow-up times of CsA and HCQ for IgAN in the included studies were less than 1 year; thus, the long-term effect is unknown. Only steroids decreased the risk of ESRD but there were significantly more SAEs. All immunosuppressants exhibited no superiority compared to glucocorticoids, whether in terms of clinical efficacy or adverse reactions.

The results of this NMA were consistent with the recent TESTING study comparing oral methylprednisolone with placebo, which showed a higher rate of remission in proteinuria and lower occurrence of ESRD in the methylprednisolone group than in the placebo group (48.2 vs. 21.8%; 2.9 vs. 7.9%) but was discontinued because of excess SAEs (14.7 vs. 3.2%) (Lv et al., 2017). Thus, the safety profiles of steroid regimens in IgAN should be considered carefully.

The use of MMF in IgAN is still controversial—this study suggested higher clinical remission in MMF monotherapy groups but no beneficial effect when the follow-up time was more than 2 years in clinical remission, ESRD, or serum creatinine level. These results were consistent with Hogg et al. (Kim et al., 2013), which was terminated early because of the lack of benefit (PR, 14 vs. 9%). Conversely, a 6-years study by Tang et al. (Tang et al., 2010) showed that patients receiving MMF have better renal survival than those receiving placebo (90 vs. 55%). These paradoxical results might be due to different follow-up times or races, as the trials were conducted in North America and Asia, respectively. Notably, the adverse reactions of MMF seem to be tolerable. The results also provided a possibility for combined therapy of MMF and low-dose glucocorticoid to reach clinical remission with fewer adverse reactions. A recent trial compared 1.5 g/d of MMF with 0.4–0.6 mg/kg·d of prednisolone against 0.8–1.0 mg/kg·d of prednisolone alone in treating IgAN (Hou et al., 2017). Although the CR rates showed no significant differences, there were fewer steroid-associated adverse events in the MMF group than the prednisone group; however, the follow-up time of this study was only 12 months. Further studies are required to confirm the long-term effects of MMF for IgAN.

We showed that TAC could be an effective treatment to induce remission in patients with IgAN within 16 weeks; however, the sample size was limited (overall 40 subjects) and the 5-years follow-up of the same cohorts showed that the anti-proteinuria effect was promptly reversed 3 months after discontinuing the drug (Yu et al., 2017). HCQ has been little studied in IgAN—a recent RCT compared the effect of a 6 months prescription of HCQ with placebo in patients with IgAN (Liu et al., 2019) and suggested that HCQ effectively reduces proteinuria and increases PR in proteinuria. In addition, HCQ was well tolerated and no SAEs were reported. As the study was an early-phase trial, the long-term renoprotective efficacy and safety still require confirmation. A recent systematic review assessed the effect of LEF in treating IgAN (Yi et al., 2019) and showed significantly lower urine protein and serum creatinine in patients treated with LEF and corticosteroids or angiotensin-converting enzyme inhibitor (ACEI) than in patients treated with corticosteroids or ACEI alone. Our results indicate that LEF monotherapy had no superiority in achieving remission of proteinuria or renal survival when compared with supportive care alone, although the direct comparison suggested that LEF might have lower proteinuria-causing activity.

Our results showed that patients with IgAN and eGFR <50 ml min^−1^·1.73 m^2^ are at a higher risk of eGFR loss, ESRD, or death owing to renal failure than those with eGFR ≥50 ml min^−1^·1.73 m^2^ when treated with steroids. This indicated that patients with IgAN and eGFR ≥50 ml min^−1^·1.73 m^2^ show a better treatment effect in preventing disease progression than those with lower eGFR; the results are consistent with those of Nagaraju et al. (Nagaraju et al., 2017). The Oxford classification of IgAN has been widely adopted in clinical practice, and it was first published in 2009 and updated in 2016 using the MEST-C scores (Mubarak, 2018). Several validation studies have reported varying results regarding the predictive value of the Oxford classification in patients with IgAN; furthermore, the correlation between MEST-C scores and immunosuppressive therapy response has not been determined (Moriyama et al., 2020). An exploratory analysis of MEST-C scores and renal outcomes in the STOP-IgAN trial showed that ESRD occurred more frequently in patients with higher T scores when additional immunosuppressants were administrated. However, the T scores did not correlate with CR or eGFR loss rate, and high M scores indicated the trend of poor CR and renal survival with no statistical significance. The E, S, and C scores were not associated with any clinical outcomes in the group administered immunosuppressants (Schimpf et al., 2018). A validation study in Japan demonstrated that corticosteroids/immunosuppressants improved renal prognosis, based on the E1, S1, and C1 scores (Moriyama et al., 2020). However, a meta-analysis based on cohort study and retrospective study showed that the M1, S1, and T1/2 scores were strongly associated with a poor response to steroid therapy (Yang et al., 2017) and progression to kidney failure (Lv et al., 2013). Our results indicated that patients with higher E scores showed better renal outcomes than those with lower E scores, but the difference was not significant. Therefore, these contradictory results could not reveal the correlation between the Oxford classification and glucocorticoid responsiveness, necessitating further research.

Several limitations of this study should be considered. First, the quality of the included trials varied, leading to significant heterogeneity. Second, the lack of reporting of main outcomes in many trials was a potential limitation. Third, some contributing studies had small sample sizes, such as the results of the funnel plot, which might have resulted in more uncertainty and less precision of the findings. Finally, the correlations between pathological lesions or other baseline characteristics and renal outcomes or immunosuppressive therapy responsiveness were not clear because of insufficient data.

## Conclusion

In conclusion, steroids might be an effective intervention strategy for IgAN to induce remission and increase renal survival; however, the adverse reactions cannot be ignored. Calcineurin inhibitors, LEF, HCQ, and MMF might improve remission of proteinuria in treating IgAN but they showed no superiority compared to steroids and the long-term effects, in particular, still require further study.

## Author Contributions

SS and TW conducted the literature retrieval, data extraction, data analyses, and manuscript writing; YL and MC assessed the risk of bias; YQ and YW designed this study, participated in the whole process. All authors approved the final manuscript.

## Funding

This work was supported by the National Natural Science Foundation of China (Grant Nos. 81403361, 81774106), and three-year projects for TCM development in Shanghai (Grant Nos. ZY (2018-2020)-RCPY-3006, ZY (2018-2020)-FWTX-4027) and project supported by Shanghai Committee of Science and Technology, China (Grant Nos.20Y21902100).

## Conflict of Interest

The authors declare that the research was conducted in the absence of any commercial or financial relationships that could be construed as a potential conflict of interest.

## Abbreviations

AZA, Azathioprine; ACEI, Angiotensin-converting enzyme inhibitor; CENTRAL, Cochrane Central Register of Controlled Trials; CYC, Cyclophosphamide; CsA, Cyclosporine; CR, Complete remission; CI, Confidence interval; eGFR, estimated glomerular filtration rate; ESRD, End-stage renal disease; HCQ, Hydroxychloroquine; IgAN, Immunoglobulin A nephropathy; KDIGO, Kidney Disease Improving Global Outcomes; LEF, Leflunomide; MMF, Mycophenolate mofetil; NMA, Network meta-analysis; PR, Partial remission; RAS, Renin-angiotensin system; RCT, Randomised controlled trials; RR, Relative risk; SMD, Standard mean differences; SUCRA, Surface under the cumulative ranking curve; SAE, Serious adverse event; TRF, Targeted-release formulation; TESTING, Therapeutic Evaluation of Steroids in IgA Nephropathy Global study; TAC, Tacrolimus.
