# Supplementary material for: Efficacy and Safety of Immunosuppressive Monotherapy Agents for IgA Nephropathy: A Network Meta-Analysis
Source: Front Pharmacol. 2021 Jan 22;11:539545. doi: 10.3389/fphar.2020.539545 (PMC7862876; doi:10.3389/fphar.2020.539545)
Supplement: Supplementary file 1 [file datasheet1.docx]

Supplementary Material

# Supplementary text 1: Search strategy

**1.1 Search strategy of** **PubMed**

#1 cyclophosphamide [MeSH Terms]

#2 cyclophosphamide [All Fields]

#3 CTX [All Fields]

#4 CYC [All Fields]

#5 azathioprine [MeSH Terms]

#6 azathioprine [All Fields]

#7 AZA [All Fields]

#8 cyclosporine [MeSH Terms]

#9 cyclosporine [All Fields]

#10 cyclosporin [All Fields]

#11 cyclosporins [MeSH Terms]

#12 cyclosporins [All Fields]

#13 cyclosporin a [All Fields]

#14 cyclosporine a [All Fields]

#15 CsA [All Fields]

#16 tacrolimus [MeSH Terms]

#17 tacrolimus [All Fields]

#18 TAC [All Fields]

#19 FK506 [All Fields]

#20 leflunomide [MeSH Terms]

#21 leflunomide [All Fields]

#22 LEF [All Fields]

#23 mycophenolate [All Fields]

#24 mycophenolic acid [MeSH Terms]

#25 mycophenolic acid [All Fields]

#26 mycophenolate mofetil [All Fields]

#27 Mycophenolate ester [All Fields]

#28 MMF [All Fields]

#29 hydroxychloroquine [MeSH Terms]

#30 hydroxychloroquine [All Fields]

#31 HCQ [All Fields]

#32 glucocorticoids [MeSH Terms]

#33 glucocorticoids [All Fields]

#34 glucocorticoid [All Fields]

#35 adrenal cortex hormones [MeSH Terms]

#36 adrenal cortex hormones [All Fields]

#37 corticosteroid [All Fields]

#38 corticosteroids [All Fields]

#39 steroids [MeSH Terms]

#40 steroids [All Fields]

#41 steroid [All Fields]

#42 prednisone [MeSH Terms]

#43 prednisone [All Fields]

#44 prednisolone [MeSH Terms]

#45 prednisolone [All Fields]

#46 methylprednisolone [MeSH Terms]

#47 methylprednisolone [All Fields]

#48 #1 OR #2 OR #3 OR #4 OR #5 OR #6 OR #7 OR #8 OR #9 OR #10 OR #11 OR #12 OR #13 OR #14 OR #15 OR #16 OR #17 OR #18 OR #19 OR #20 OR #21 OR #22 OR #23 OR #24 OR #25 OR #26 OR #27 OR #28 OR #29 OR #30 OR #31 OR #32 OR #33 OR #34 OR #35 OR #36 OR #37 OR #38 OR #39 OR #40 OR #41 OR #42 OR #43 OR #44 OR #45 OR #46 OR #47

#49 glomerulonephritis, iga [MeSH Terms]

#50 iga nephropathy [All Fields]

#51 iga glomerulonephritis [All Fields]

#52 glomerulonephritis iga [All Fields]

#53 immunoglobulin a nephropathy [All Fields]

#54 IgAN [All Fields]

#55 iga nephritis

#56 berger's disease [All Fields]

#57 #49 OR #50 OR #51 OR #52 OR #53 OR #54 OR #55 OR #56

#58 random* [All Fields]

#59 randomized controlled trial [Publication Type]

#60 randomized controlled trials as topic [MeSH Terms]

#61 randomized controlled trial [All Fields]

#62 randomised controlled trial [All Fields]

#63 randomized trial [All Fields]

#64 random allocation [MeSH Terms]

#65 random allocation [All Fields]

#66 clinical trials as topic [MeSH Terms]

#67 clinical trial [All Fields]

#68 clinical trial [Publication Type]

#69 controlled clinical trial [Publication Type]

#70 controlled clinical trial [All Fields]

#71 controlled clinical trials as topic [MeSH Terms]

#72 controlled trial [All Fields]

#73 trial [All Fields]

#74 RCT [All Fields]

#75 #58 OR #59 OR #60 OR #61 OR #62 OR #63 OR #64 OR #65 OR #66 OR #67 OR #68 OR #69 OR #70 OR #71 OR #72 OR #73 OR #74

#76 #48 AND #57 AND #75

**1.2 Search strategy of** **Embase**

#1 'immunoglobulin a nephropathy'/exp

#2 iga AND nephropathy

#3 iga AND glomerulonephritis

#4 glomerulonephritis AND iga

#5 immunoglobulin AND a AND nephropathy

#6 IgAN

#7 #1 OR #2 OR #3 OR #4 OR #5 OR #6

#8 'clinical trial'/exp

#9 'controlled clinical trial'/exp

#10 'randomized controlled trial'/exp

#11 'randomized controlled trial':it

#12 'controlled clinical trial':it

#13 'clinical trial':it

#14 randomised AND controlled AND trial

#15 randomized AND trial

#16 random AND allocation

#17 clinical AND trial

#18 controlled AND clinical AND trial

#19 controlled AND trial

#20 trial

#21 RCT

#22 #8 OR #9 OR #10 OR #11 OR #12 OR #13 OR #14 OR #15 OR #16 OR #17 OR #18 OR #19 OR #20 OR #21

#23 'cyclophosphamide'/exp

#24 'azathioprine'/exp

#25 'cyclosporine'/exp

#26 'tacrolimus'/exp

#27 'leflunomide'/exp

#28 'mycophenolate mofetil'/exp

#29 'hydroxychloroquine'/exp

#30 'glucocorticoid'/exp

#31 'corticosteroid'/exp

#32 'steroid'/exp

#33 'prednisone'/exp

#34 'prednisolone'/exp

#35 'methylprednisolone'/exp

#36 mycophenolate

#37 mycophenolic AND acid

#38 mycophenolate AND mofetil

#39 mycophenolate AND ester

#40 MMF

#41 hydroxychloroquine

#42 HCQ

#43 glucocorticoids

#44 glucocorticoid

#45 adrenal AND cortex AND hormones

#46 corticosteroid

#47 corticosteroids

#48 steroids

#49 steroid

#50 prednisone

#51 prednisolone

#52 methylprednisolone

#53 cyclophosphamide

#54 CTX

#55 CYC

#56 azathioprine

#57 AZA

#58 cyclosporine

#59 cyclosporin

#60 cyclosporins

#61 cyclosporin AND a

#62 cyclosporine AND a

#63 CsA

#64 tacrolimus

#65 TAC

#66 FK506

#67 leflunomide

#68 LEF

# 69 #23 OR #24 OR #25 OR #26 OR #27 OR #28 OR #29 OR #30 OR #31 OR #32 OR #33 OR #34 OR #35 OR #36 OR #37 OR #38 OR #39 OR #40 OR #41 OR #42 OR #43 OR #44 OR #45 OR #46 OR #47 OR #48 OR #49 OR #50 OR #51 OR #52 OR #53 OR #54 OR #55 OR #56 OR #57 OR #58 OR #59 OR #60 OR #61 OR #62 OR #63 OR #64 OR #65 OR #66 OR #67 OR #68

#70 #7 AND #22 AND #69

**1.3 Search strategy in Web of Science**

#1 TOPIC: (glomerulonephritis iga)

#2 TOPIC: (iga nephropathy)

#3 TOPIC: (iga glomerulonephritis)

#4 TOPIC: (immunoglobulin a nephropathy)

#5 TOPIC: (IgAN)

#6 TOPIC: (berger's disease)

#7 ALL FIELDS: (glomerulonephritis iga)

#8 ALL FIELDS: (iga nephropathy)

#9 ALL FIELDS: (iga glomerulonephritis)

#10 ALL FIELDS: (immunoglobulin a nephropathy)

#11 ALL FIELDS: (IgAN)

#12 ALL FIELDS: (berger's disease)

#13 #1 OR #2 OR #3 OR #4 OR #5 OR #6 OR #7 OR #8 OR #9 OR #10 OR #11 OR #12

#14 ALL FIELDS: (randomized controlled trial)

#15 ALL FIELDS: (randomised controlled trial)

#16 ALL FIELDS: (randomized trial)

#17 ALL FIELDS: (random allocation)

#18 ALL FIELDS: (clinical trial)

#19 ALL FIELDS: (controlled clinical trial)

#20 ALL FIELDS: (controlled trial)

#21 ALL FIELDS: (trial)

#22 ALL FIELDS: (controlled clinical trial)

#23 ALL FIELDS: (RCT)

#24 TOPIC: (randomized controlled trial)

#25 TOPIC: (randomised controlled trial)

#26 TOPIC: (randomized trial)

#27 TOPIC: (random allocation)

#28 TOPIC: (clinical trial)

#29 TOPIC: (controlled clinical trial)

#30 TOPIC: (controlled trial)

#31 TOPIC: (trial)

#32 TOPIC: (controlled clinical trial)

#33 TOPIC: (RCT)

#34 #14 OR #15 OR #16 OR #17 OR #18 OR #19 OR #20 OR #21 OR #22 OR #23 OR #24 OR #25 OR #26 OR #27 OR #28 OR #29 OR #30 OR #31 OR #32 OR #33

#35 ALL FIELDS: (cyclophosphamide)

#36 ALL FIELDS: (CTX)

#37 ALL FIELDS: (CYC)

#38 ALL FIELDS: (azathioprine)

#39 ALL FIELDS: (AZA)

#40 ALL FIELDS: (cyclosporine)

#41 ALL FIELDS: (cyclosporin)

#42 ALL FIELDS: (cyclosporins)

#43 ALL FIELDS: (CsA)

#44 ALL FIELDS: (tacrolimus)

#45 ALL FIELDS: (TAC)

#46 ALL FIELDS: (FK506)

#47 ALL FIELDS: (leflunomide)

#48 ALL FIELDS: (LEF)

#49 ALL FIELDS: (mycophenolate)

#50 ALL FIELDS: (mycophenolic acid)

#51 ALL FIELDS: (mycophenolate mofetil)

#52 ALL FIELDS: (Mycophenolate ester)

#53 ALL FIELDS: (MMF)

#54 ALL FIELDS: (hydroxychloroquine)

#55 ALL FIELDS: (HCQ)

#56 ALL FIELDS: (glucocorticoid)

#57 ALL FIELDS: (steroid)

#58 ALL FIELDS: (corticosteroid)

#59 ALL FIELDS: (adrenal cortex hormone)

#60 ALL FIELDS: (corticosteroids)

#61 ALL FIELDS: (glucocorticoids)

#62 ALL FIELDS: (steroids)

#63 ALL FIELDS: (prednisone)

#64 ALL FIELDS: (prednisolone)

#65 ALL FIELDS: (methylprednisolone)

#66 TOPIC: (cyclophosphamide)

#67 TOPIC: (CTX)

#68 TOPIC: (CYC)

#69 TOPIC: (azathioprine)

#70 TOPIC: (AZA)

#71 TOPIC: (cyclosporine)

#72 TOPIC: (cyclosporin)

#73 TOPIC: (cyclosporins)

#74 TOPIC: (CsA)

#75 TOPIC: (tacrolimus)

#76 TOPIC: (TAC)

#77 TOPIC: (FK506)

#78 TOPIC: (leflunomide)

#79 TOPIC: (LEF)

#80 TOPIC: (mycophenolate)

#81 TOPIC: (mycophenolic acid)

#82 TOPIC: (mycophenolate mofetil)

#83 TOPIC: (Mycophenolate ester)

#84 TOPIC: (MMF)

#85 TOPIC: (hydroxychloroquine)

#86 TOPIC: (HCQ)

#87 TOPIC: (glucocorticoid)

#88 TOPIC: (corticosteroid)

#89 TOPIC: (steroid)

#90 TOPIC: (adrenal cortex hormone)

#91 TOPIC: (corticosteroids)

#92 TOPIC: (glucocorticoids)

#93 TOPIC: (steroids)

#94 TOPIC: (prednisone)

#95 TOPIC: (prednisolone)

#96 TOPIC: (methylprednisolone)

#97 #35 OR #36 OR #37 OR #38 OR #39 OR #40 OR #41 OR #42 OR #43 OR #44 OR #45 OR #46 OR #47 OR #48 OR #49 OR #50 OR #51 OR #52 OR #53 OR #54 OR #55 OR #56 OR #57 OR #58 OR #59 OR #60 OR #61 OR #62 OR #63 OR #64 OR #65 OR #66 OR #67 OR #68 OR #69 OR #70 OR #71 OR #72 OR #73 OR #74 OR #75 OR #76 OR #77 OR #78 OR #79 OR #80 OR #81 OR #82 OR #83 OR #84 OR #85 OR #86 OR #87 OR #88 OR #89 OR #90 OR #91 OR #92 OR #93 OR #94 OR #95 OR #96

#98 #13 AND #34 AND #97

**1.4 Search strategy of** **CENTRAL**

#1 MeSH descriptor: [Glomerulonephritis, IGA] explode all trees

#2 iga nephropathy

#3 iga glomerulonephritis

#4 immunoglobulin a nephropathy

#5 berger's disease

#6 IgAN (Word variations have been searched)

#7 #1 OR #2 OR #3 OR #4 OR #5 OR #6

#8 (randomized controlled trial):pt

#9 randomized controlled trial

#10 randomized trial

#11 clinical trial

#12 controlled clinical trial

#13 (controlled clinical trial):pt

#14 random allocation

#15 controlled trial

#16 trial

#17 RCT (Word variations have been searched)

#18 #8 OR #9 OR #10 OR #11 OR #12 OR #13 OR #14 OR #15 OR #16 OR #17

#19 (cyclophosphamide):ti,ab,kw

#20 (azathioprine):ti,ab,kw

#21 (cyclosporine):ti,ab,kw

#22 (cyclosporin):ti,ab,kw

#23 (cyclosporins):ti,ab,kw

#24 (cyclosporin a):ti,ab,kw

#25 (cyclosporine a):ti,ab,kw

#26 (tacrolimus):ti,ab,kw

#27 (leflunomide):ti,ab,kw

#28 (mycophenolate):ti,ab,kw

#29 (mycophenolic acid):ti,ab,kw

#30 (mycophenolate mofetil):ti,ab,kw

#31 (Mycophenolate ester):ti,ab,kw

#32 (hydroxychloroquine):ti,ab,kw

#33 (HCQ):ti,ab,kw

#34 (CTX):ti,ab,kw

#35 (CYC):ti,ab,kw

#36 (AZA):ti,ab,kw

#37 (CsA):ti,ab,kw

#38 (TAC):ti,ab,kw

#39 (FK506):ti,ab,kw

#40 (LEF):ti,ab,kw

#41 (MMF):ti,ab,kw

#42 (glucocorticoid):ti,ab,kw

#43 (corticosteroid):ti,ab,kw

#44 (steroid):ti,ab,kw

#45 (prednisone):ti,ab,kw

#46 (prednisolone):ti,ab,kw

#47 (methylprednisolone):ti,ab,kw

#48 (adrenal cortex hormone):ti,ab,kw (Word variations have been searched)

#49 #19 OR #20 OR #21 OR #22 OR #23 OR #24 OR #25 OR #26 OR #27 OR #28 OR #29 OR #30 OR #31 OR #32 OR #33 OR #34 OR #35 OR #36 OR #37 OR #38 OR #39 OR #40 OR #41 OR #42 OR #43 OR #44 OR #45 OR #46 OR #47 OR #48

#50 #7 AND #18 AND #49

# Supplementary Tables

**Supplementary Table S1: PRISMA checklist**

| **Section/Topic** | **Item #** | **Checklist Item** | **Reported on Page #** |
| --- | --- | --- | --- |
| **TITLE** |  |  |  |
| Title | 1 | Identify the report as a systematic review *incorporating a network meta-analysis (or related form of meta-analysis).* | *1* |
|  |  |  |  |
| **ABSTRACT** |  |  |  |
| Structured summary | 2 | Provide a structured summary including, as applicable:  **Background:** main objectives  **Methods:** data sources; study eligibility criteria, participants, and interventions; study appraisal; and *synthesis methods, such as network meta-analysis.*  **Results:** number of studies and participants identified; summary estimates with corresponding confidence/credible intervals; *treatment rankings may also be discussed. Authors may choose to summarize pairwise comparisons against a chosen treatment included in their analyses for brevity.*  **Discussion/Conclusions:** limitations; conclusions and implications of findings.  **Other:** primary source of funding; systematic review registration number with registry name. | 1 |
|  |  |  |  |
| **INTRODUCTION** |  |  |  |
| Rationale | 3 | Describe the rationale for the review in the context of what is already known*, including mention of why a network meta-analysis has been conducted.* | *2* |
| Objectives | 4 | Provide an explicit statement of questions being addressed, with reference to participants, interventions, comparisons, outcomes, and study design (PICOS). | 2 |
|  |  |  |  |
| **METHODS** |  |  |  |
| Protocol and registration | 5 | Indicate whether a review protocol exists and if and where it can be accessed (e.g., Web address); and, if available, provide registration information, including registration number. | 2 |
| Eligibility criteria | 6 | Specify study characteristics (e.g., PICOS, length of follow-up) and report characteristics (e.g., years considered, language, publication status) used as criteria for eligibility, giving rationale. *Clearly describe eligible treatments included in the treatment network, and note whether any have been clustered or merged into the same node (with justification).* | *2-3* |
| Information sources | 7 | Describe all information sources (e.g., databases with dates of coverage, contact with study authors to identify additional studies) in the search and date last searched. | 2 |
| Search | 8 | Present full electronic search strategy for at least one database, including any limits used, such that it could be repeated. | 2 |
| Study selection | 9 | State the process for selecting studies (i.e., screening, eligibility, included in systematic review, and, if applicable, included in the meta-analysis). | 3 |
| Data collection process | 10 | Describe method of data extraction from reports (e.g., piloted forms, independently, in duplicate) and any processes for obtaining and confirming data from investigators. | 3 |
| Data items | 11 | List and define all variables for which data were sought (e.g., PICOS, funding sources) and any assumptions and simplifications made. | 3 |
| **Geometry of the network** | **S1** | Describe methods used to explore the geometry of the treatment network under study and potential biases related to it. This should include how the evidence base has been graphically summarized for presentation, and what characteristics were compiled and used to describe the evidence base to readers. | *4* |
| Risk of bias within individual studies | 12 | Describe methods used for assessing risk of bias of individual studies (including specification of whether this was done at the study or outcome level), and how this information is to be used in any data synthesis. | 3 |
| Summary measures | 13 | State the principal summary measures (e.g., risk ratio, difference in means). *Also describe the use of additional summary measures assessed, such as treatment rankings and surface under the cumulative ranking curve (SUCRA) values, as well as modified approaches used to present summary findings from meta-analyses.* | 3 |
| Planned methods of analysis | 14 | Describe the methods of handling data and combining results of studies for each network meta-analysis. This should include, but not be limited to:   - *Handling of multi-arm trials;* - *Selection of variance structure;* - *Selection of prior distributions in Bayesian analyses; and* - *Assessment of model fit.* | 3 |
| **Assessment of Inconsistency** | **S2** | Describe the statistical methods used to evaluate the agreement of direct and indirect evidence in the treatment network(s) studied. Describe efforts taken to address its presence when found. | 3 |
| Risk of bias across studies | 15 | Specify any assessment of risk of bias that may affect the cumulative evidence (e.g., publication bias, selective reporting within studies). | 3 |
| Additional analyses | 16 | Describe methods of additional analyses if done, indicating which were pre-specified. This may include, but not be limited to, the following:   - Sensitivity or subgroup analyses; - Meta-regression analyses; - *Alternative formulations of the treatment network; and* - *Use of alternative prior distributions for Bayesian analyses (if applicable).* |  |
|  |  |  |  |
| **RESULTS†** |  |  |  |
| Study selection | 17 | Give numbers of studies screened, assessed for eligibility, and included in the review, with reasons for exclusions at each stage, ideally with a flow diagram. | 4 |
| **Presentation of network structure** | **S3** | Provide a network graph of the included studies to enable visualization of the geometry of the treatment network. | *4* |
| **Summary of network geometry** | **S4** | Provide a brief overview of characteristics of the treatment network. This may include commentary on the abundance of trials and randomized patients for the different interventions and pairwise comparisons in the network, gaps of evidence in the treatment network, and potential biases reflected by the network structure. | *4* |
| Study characteristics | 18 | For each study, present characteristics for which data were extracted (e.g., study size, PICOS, follow-up period) and provide the citations. | 4 |
| Risk of bias within studies | 19 | Present data on risk of bias of each study and, if available, any outcome level assessment. | 4 |
| Results of individual studies | 20 | For all outcomes considered (benefits or harms), present, for each study: 1) simple summary data for each intervention group, and 2) effect estimates and confidence intervals. *Modified approaches may be needed to deal with information from larger networks.* | *4* |
| Synthesis of results | 21 | Present results of each meta-analysis done, including confidence/credible intervals. *In larger networks, authors may focus on comparisons versus a particular comparator (e.g. placebo or standard care), with full findings presented in an appendix. League tables and forest plots may be considered to summarize pairwise comparisons.* If additional summary measures were explored (such as treatment rankings), these should also be presented. | *4-5* |
| **Exploration for inconsistency** | **S5** | Describe results from investigations of inconsistency. This may include such information as measures of model fit to compare consistency and inconsistency models, *P* values from statistical tests, or summary of inconsistency estimates from different parts of the treatment network. | *4-5* |
| Risk of bias across studies | 22 | Present results of any assessment of risk of bias across studies for the evidence base being studied. | 4 |
| Results of additional analyses | 23 | Give results of additional analyses, if done (e.g., sensitivity or subgroup analyses, meta-regression analyses*, alternative network geometries studied, alternative choice of prior distributions for Bayesian analyses,* and so forth). | *5* |
|  |  |  |  |
| **DISCUSSION** |  |  |  |
| Summary of evidence | 24 | Summarize the main findings, including the strength of evidence for each main outcome; consider their relevance to key groups (e.g., healthcare providers, users, and policy-makers). | 5-6 |
| Limitations | 25 | Discuss limitations at study and outcome level (e.g., risk of bias), and at review level (e.g., incomplete retrieval of identified research, reporting bias). *Comment on the validity of the assumptions, such as transitivity and consistency. Comment on any concerns regarding network geometry (e.g., avoidance of certain comparisons).* | 6 |
| Conclusions | 26 | Provide a general interpretation of the results in the context of other evidence, and implications for future research. | 7 |
|  |  |  |  |
| **FUNDING** |  |  |  |
| Funding | 27 | Describe sources of funding for the systematic review and other support (e.g., supply of data); role of funders for the systematic review. This should also include information regarding whether funding has been received from manufacturers of treatments in the network and/or whether some of the authors are content experts with professional conflicts of interest that could affect use of treatments in the network. | *7* |

PICOS = population, intervention, comparators, outcomes, study design.

* Text in italics indicateS wording specific to reporting of network meta-analyses that has been added to guidance from the PRISMA statement.

† Authors may wish to plan for use of appendices to present all relevant information in full detail for items in this section.

**Supplementary Table S2: Characteristics of eligible studies**

| Study | Location | Main included criteria | Treatment; Duration | Control | Supportive care | Follow-up | Treatment  N (M/F); Age(years) | Control  N (M/F); Age(years) | Outcome |
| --- | --- | --- | --- | --- | --- | --- | --- | --- | --- |
| Lai 1986 | China | Serum albuminemia <35g/L, proteinuria >3.5g/d | Initial prednisolone/prednisone 40-60mg/day; four months | No steroid | Not mentioned | 37.7 ± 28.1/38.7 ± 19.4 months | 17 (10/7); 28.9 ± 7.9 | 17 (7/10); 26.9 ± 8.6 | CR; UPE; SCr; SAE |
| Lai 1987 | China | Proteinuria ≥ 1.5g/d; creatinine clearance > 50ml/min/1.73m^2^ | Initial cyclosporin 5mg/kg/day, maintain a concentration of 45-150ug/l; 12 weeks | Placebo | Blood pressures < 150/90mmHg, treated by nadolol | 24 weeks | 9(4/5); 33.1 ± 4.2 | 10(6/4); 38.7 ± 12.97 | PR; UPE; SCr; SAE |
|  |  |  |  |  |  |  |  |  |  |
| Julian 1993 | America | Creatinine clearance > 25 ml/min/1.73m^2^ | Initial alternate day prednisone 60 mg; 2years | No steroid | Diastolic blood pressure < 95 mmHg | 6-24 months | 17; 9 woman 34 ± 12.4; 26 man 39 ± 12.7 | 18 | UPE; SCr; SAE |
| Pozzi 1999; Locatelli 2001; Pozzi 2004 | Italy | Proteinuria 1·0 - 3·5 g/d, and SCr ≤ 133mol/L (1.5 mg/dL) | Methylprednisolone 1g intravenously for 3 consecutive days; prednisone 0·5 mg/kg on alternate days; 6 months | No steroid | Diuretics, antihypertensive drugs (ACEIs) and antiplatelet agents | 10 years | 43(30/13); 26 - 45 | 43(31/12); 29 - 51 | CR; UPE; ESRD; SAE |
| Shoji 2000 | Japan | Proteinuria <1.5 g/d, SCr < 1.5 mg/dL | Initial prednisolone 0.8 mg/kg; 1 year | Dipyridamole 300mg/d | Blood pressure <130/85 mmHg | 13.4 ± 2.4 months | 11(5/6); 28.7 ± 11.2 | 8(1/7); 33.3 ± 11.9 | UPE; SCr; SAE |
| Chen 2002 | China | Lee’s grade IV - V, proteinuria ≥ 2.0g/d, and SCr ≤ 4 mg/dL | MMF 1.0g/d (< 50kg) or 1.5g/d (> 50kg); 18months | Prednisone 0.8mg/kg/d | Blood pressure < 130/80 mmHg | 18 months | 31(25/6); 28 ± 10 | 31(22/9); 29 ± 10 | CR; PR; UPE; SAE |
| Katafuchi 2003 | Japan | Moderate histological characteristics, SCr ≤ 133mol/L (1.5 mg/dL) | Initial prednisolone 20 mg/d; 24months | No steroid | Dipyridamole 150 or 300 mg/d | 65 ± 25/64 ± 23 months | 43(15/28); 33.6 ± 13.4 | 47(22/25); 32.5 ± 10.8 | UPE; SCr; ESRD; SAE; PR |
| Lee 2003 | Koren | Proteinuria >1.0 g/d, SCr < 2.0mg/dL | Initial prednisolone 1mg/kg/day; 1year | No steroid | ARB | 15.4 ± 3.5/19.8 ± 7.4 months | 18(12/6); 36 ± 18 | 20(10/10); 31 ± 20 | CR; UPE; SCr |
| Maes 2004 | Belgium | Inulin clearance >20 but <70 mL/min/1.73 m^2^ | MMF 1g twice a day; 36months | Placebo | Restriction of salt intake (< 5 g NaCl/day), ACE inhibitors (blood pressure ≤125/75 mm Hg) | 36 months | 21(16/5); 39 ± 11 | 13(8/5); 43 ± 15 | UPE; SCr; ESRD; SAE |
| Frisch 2005 | America | Proteinuria ≥1g/d | MMF 1.0g twice a day; 12months | Placebo | Blood pressure ≤ 130/80 mmHg, treated by ACEI/ARB | 2 years | 17(16/1); 19 - 72 | 15(11/4); 22 - 59 | PR; ESRD; SAE; SCr; UPE |
| Kim 2005 | Koren | Proteinuria >1.0 g/d, SCr <1.3 mg/dL | Initial prednisolone 1mg/kg/day; 1year | No steroid | ARB | 28.0 ± 6.0/30.3 ± 5.9months | 12(5/7); 32.3 ± 8.4 | 11(5/6); 33.7 ± 10.4 | CR; UPE; SCr |
| Tang 2005; Tang 2010 | China | Proteinuria >1.0 g/d | MMF 2 g/d (≥ 60 kg), or 1.5 g/d (<60 kg); 6months | No MMF | Sodium-restricted diet; blood pressure < 125/85 mmHg treated by ACEI/ARB | 72 weeks | 20(6/14); 42.1 ± 2.6 | 20(8/12); 43.3 ± 2.8 | CR; PR; SAE; ESRD; UPE |
| Hogg 2006 | America | eGFR ≥50 ml/min/1.73 m^2^; persistent severe proteinuria | Initial alternate-day prednisone 60 mg/m^2^; 24months | Placebo | Enalapril | 2 years | 33(23/10); 24 ± 10 | 31(20/11); 21 ± 10 | PR; SAE |
| Lou 2006 | China | Proteinuria 1.0 – 3.0g/d; SCr < 354 μmol/L | Leflunomide 60 mg/d for 3 days, then 20 mg/d; 6months | Fosinopril | Blood pressure < 125/75 mmHg | 7 months | 24(8/16); 29 ± 11 | 22(10/12); 34 ± 11 | CR; PR; SAE |
| Koike 2008 | China | Mild histological activities | Initial prednisolone 0.4 mg/kg/d; 24 months. | No steroid | Anti-platelet drugs; ACEI for hypertensive patients. | 24 months | 24(6/18); 37.9 ± 10.1 | 24(5/19); 38.3 ± 12.7 | UPE |
| Lv 2009 | China | Proteinuria 1 - 5 g/d and eGFR > 30 mL/min/1.73 m^2^ | Initial prednisone 0.8 to 1.0 mg/kg/d; 6 - 8 months | No steroid | Cilazapril 5 mg/d. | 26 ± 8/28 ± 7 months | 33(20/13); 27.8 ± 8.9 | 30(19/11); 30.4 ± 8.8 | PR; UPE; ESRD; SAE |
| Manno 2009 | Italy | Proteinuria ≥1.0 g/d, and eGFR ≥ 50 ml/min/1.73 m^2^ | Initial prednisone 1.0 mg/kg/d (maximum 75 mg/day); 6months | No steroid | Blood pressure < 120/80 mmHg treated by ramipril | 45.3 ± 83.4/31.4 ± 77.2 months | 48(33/15); 31.8 ± 11.3 | 49(35/14); 34.9 ± 11.2 | PR; ESRD; SAE |
| Kim 2013, Yu 2017 | Korea | SCr ≤1.5 mg/dL or eGFR ≥ 45 ml/min/1.73 m^2^, UACR 0.3 - 3.0 g/g | Initial tacrolimus 0.1 mg/kg/d, maintain trough levels at 5 - 10 ng/ml; 16weeks | Placebo | ARB for hypertensive patients | 57.9 ± 13.8 months | 20(6/14); 36.9 ± 11.4 | 20(6/14); 40.1 ± 12.8 | PR; SCr; UPE; SAE |
| Cheng 2015 | China | Lee’s histological grade II - IV; proteinuria 0.5 -3.5 g/d; SCr < 3 mg/dL | Leflunomide 20mg/day | Placebo | Valsartan 80 mg/d, and/or clopidogrel 75 mg/d | 24months | 84; 33.33 ± 8.78 | 84; 33.98 ± 9.70 | SCr; UPE; ESRD; SAE |
|  |  |  |  |  |  |  |  |  |  |
| Hogg 2015 | America, Canada | UPCR ≥ 0.6 g/g (males) or ≥ 0.8 g/g (females), and Egfr ≥ 40 mL/min/ 1.73 m^2^ | MMF 25 to 36 mg/kg/d ( maximum 1 g twice a day); 12 months | Placebo | Omacor 1 g/d, and lisinopril (or losartan) | 6 - 24months | 25(14/11); 31.8 ± 11.7 | 27(18/9); 32.2 ± 13.2 | CR; PR; SAE |
| Wu 2016 | China | Lee’s grade II - IV, proteinuria 0.5 - 3.5 g/d, SCr < 265 μmol/L | Leflunomide 20mg/day | Placebo | Blood pressure ≤ 130/80 mmHg | 24 weeks | 199(116/83); 37.59 ± 10.53 | 200(115/85); 37.77 ± 9.74 | SAE |
| Fellstrom 2017 | Ten European countries | eGFR ≥ 45 mL/min/ 1.73 m^2^ and UPCR > 0·5g/g or Proteinuria ≥ 0·75 g/day | Targeted-release formulation of budesonide (16 mg/d and 8 mg/d); 9months | Placebo | Blood pressure <130/80mmHg treated by ACEI/ARB | 12 months | 99(70/29); 39.1 ± 12.51 | 50(35/15); 38.9 ± 12.0 | ESRD; SAE |
|  |  |  |  |  |  |  |  |  |  |
| Lv 2017 | China, Australia, India, Canada, and Malaysia | eGFR 20 - 120 mL/min/1.73m^2^, and proteinuria > 1 g/d | Initial methylprednisolone 0.6 to 0.8 mg/kg/d; 6 – 8 minths | Placebo | Blood pressure control and ACEI/ARB | 5years | 136(86/50); 38.6 ± 11.5 | 126(80/46); 38.6 ± 10.7 | PR; UPE; ESRD; SAE |
|  |  |  |  |  |  |  |  |  |  |
| Tang 2018 | China | Proteinuria 0.5 - 1.0 g/d; and eGFR ≥ 90 ml/min/1.73 m^2^ | Initial methylprednisolone 1 mg/kg/d (maximum 60 mg/d), gradually decreased | No steroid | Lotensin 10 mg/d and/or Losartan 50mg/d, blood pressure ≤ 130/80 mmHg. | 3years | 22(12/10); 35.12 ± 6.10 | 23(11/12); 34.50 ± 7.10 | UPE; SAE |
| Liu 2019 | China | eGFR > 30 mL/ min/1.73m^2^, and proteinuria 0.75 - 3.5 g/d | HCQ 0.2 g twice daily, 0.1 g 3 times daily , and 0.1 g twice daily for patients of CKD 2 stage, 3a stage, and 3b stage, respectively | Placebo | A maximum or tolerable dose of ACEI/ARB | 6 months | 30(19/11); 37.6 ± 11.6 | 30(20/10); 35.6 ± 9.6 | PR; UPE; SAE |

N = number of participants; M = male; F = female; eGFR = estimated glomerular filtration rate; ACEI = angiotensin-converting enzyme inhibitor; ARB = angiotensin receptor antagonist; UACR = urine albumin to creatinine ratio; UPCR = urine proteinuria to creatinine ratio; MMF = mycophenolate mofetil; HCQ = hydroxychloroquine; CKD = chronic kidney disease; CR = complete remission; PR = partial remission; ESRD = end-stage renal disease; SAE = serious adverse event; UPE = urinary protein excretion; SCr = serum creatinine.

**Supplementary Table S3: Results of pairwise meta-analysis for urinary protein excretion**

|  | N | *P* value for heterogeneity | SMD (95% CIs) |
| --- | --- | --- | --- |
| Hydroxychloroquine vs. Control | 1 | - | **-1.09 (-1.64, -0.55)** |
| Leflunomide vs. Control | 1 | - | **-0.58 (-0.89, -0.27)** |
| Cyclosporine vs. Control | 1 | - | 0.13 (-0.78, 1.03) |
| Tacrolimus vs. Control | 1 | - | 0.52 (-0.11, 1.15) |
| Mycophenolate mofetil vs. Control | 3 | 0.04 | -0.18 (-0.88, 0.51) |
| Mycophenolate mofetil vs. Steroids | 1 | - | **-0.77 (-1.28, -0.25)** |
| Steroids vs. Control | 11 | 0.002 | **-0.69 (-0.98, -0.41)** |

N = number of studies; SMD = standardized mean difference; CI = confidence interval. Statistical significance is defined as 95% CIs that do not overlap zero (bold text).

**Supplementary Table S4: Results of meta-analysis for serum creatinine level**

| Leflunomide |  |  |  |  | -0.25 (-0.56,0.05), N = 1 |
| --- | --- | --- | --- | --- | --- |
| -0.38 (-1.33, 0.57) | Cyclosporine |  |  |  | 0.13 (-0.77, 1.03), N = 1 |
| -0.62 (-1.31, 0.08) | -0.23 (-1.33, 0.86) | Tacrolimus |  |  | 0.37 (-0.25,0.99), N = 1 |
| -0.54 (-1.12, 0.04) | -0.16 (-1.18, 0.87) | 0.08 (-0.72, 0.87) | Mycophenolate mofetil |  | 0.29 (-0.19,0.78), N = 2, *P* = 0.65 |
| -0.11 (-0.53, 0.30) | 0.27 (-0.67, 1.22) | 0.50 (-0.18, 1.19) | 0.43 (-0.14, 1.00) | Steroids | -0.14 (-0.42, 0.14), N = 6, *P* = 0.87 |
| -0.25 (-0.56, 0.05) | 0.13 (-0.77, 1.03) | 0.36 (-0.26, 0.99) | 0.29 (-0.21, 0.78) | -0.14 (-0.42, 0.14) | Control |

The results of network meta-analysis (bottom left) and pairwise meta-analysis (upper right) for serum creatinine level. Estimates are shown as standard mean difference (95% confidence intervals). The comparison estimate is for the column-defining treatment versus the row-defining treatment. Statistical significance is defined as 95% confidence intervals that do not overlap zero. N = number of studies; *P* = p-value for heterogeneity.

**Supplementary Table S5: Sensitivity analysis for follow-up of more than two years**

| End-stage renal disease | | | | |
| --- | --- | --- | --- | --- |
| Leflunomide | 0.11 (0.00, 3.36) | 0.26 (0.01, 7.61) | 0.09 (0.00, 2.24) |  |
| - | Mycophenolate mofetil | 2.50 (0.46, 13.67) | 0.86 (0.23, 3.25) |  |
| - | 0.96 (0.27, 3.46) | Steroids | **0.35 (0.12, 0.98)** |  |
| - | 1.41 (0.40, 4.92) | **1.47 (1.10, 1.96)** | Control |  |
| Clinical remission | | | | |

Estimates are shown as relative risk (95% confidence interval [CI]). The risk estimate is for the column-defining treatment compared to the row-defining treatment. Statistical significance is defined as 95% CIs that do not overlap one (bold text).

**Supplementary Table S6: Sensitivity analysis for participants more than 100**

| End-stage renal disease | | | |
| --- | --- | --- | --- |
| Leflunomide | 0.11 (0.00, 3.36) | 0.26 (0.01, 7.61) | 0.09 (0.00, 2.24) |
| - | Mycophenolate mofetil | 2.50 (0.46, 13.67) | 0.86 (0.23, 3.25) |
| - | 1.36 (0.75, 2.48) | Steroids | **0.35 (0.12, 0.98)** |
| - | **2.05 (1.15, 3.65)** | **1.50 (1.17, 1.93)** | Control |
| Clinical remission | | | |

Estimates are shown as relative risk (95% confidence interval [CI]). The risk estimate is for the column-defining treatment compared to the row-defining treatment. Statistical significance is defined as 95% CIs that do not overlap one (bold text).
